# Single Cell and Machine Learning–Based Analysis of Lysosome Mediated Cell Death Reveals Novel Prognostic Biomarkers and Therapeutic Targets in Cervical Cancer

**DOI:** 10.1155/ijog/8487184

**Published:** 2026-05-30

**Authors:** Yurong Cheng, Xuan Li, Dong Yan

**Affiliations:** ^1^ Department of Oncology, Beijing Luhe Hospital, Capital Medical University, Beijing, China, luhehospital.com; ^2^ Department of Obstetrics and Gynecology, Beijing Luhe Hospital, Capital Medical University, Beijing, China, luhehospital.com

**Keywords:** cervical cancer, lysosome-mediated cell death, machine learning, prognostic biomarkers, single-cell RNA sequencing

## Abstract

**Background:**

Cervical cancer is among the most prevalent gynecological malignancies globally, and its inherent molecular heterogeneity remains a persistent obstacle to uniform treatment efficacy and patient survival. Lysosome‐mediated cell death (LDCD) has recently emerged as a mechanistically distinct form of regulated cell death with growing relevance in tumor biology, influencing both oncogenic progression and responsiveness to therapy. Nevertheless, its functional significance in cervical cancer and its interactions with the tumor microenvironment (TME) at single‐cell resolution remain insufficiently characterized.

**Methods:**

We conducted an integrative analysis combining single‐cell RNA sequencing (scRNA‐seq) with 15 machine learning algorithms to examine LDCD‐related gene expression patterns in cervical cancer. Multicohort transcriptomic data were obtained from TCGA and GEO databases, and scRNA‐seq data were retrieved from the GSE138080 dataset. Core hub genes were identified through differential expression analysis, protein–protein interaction network construction, and consensus clustering. Prognostic models were built using multiple machine learning frameworks and evaluated by concordance index. Immune cell infiltration was quantified via CIBERSORT, and single‐cell‐level validation was performed with the Seurat pipeline.

**Results:**

Six LDCD‐associated hub genes—CTSV, FER, GGA2, LAMP3, STAB1, and STAB2—were identified as significant prognostic determinants. Univariate Cox regression demonstrated that CTSV (*p* = 0.048) and STAB2 (*p* = 0.017) were independently associated with patient survival. Consensus clustering stratified patients into two molecularly distinct subtypes with markedly different survival trajectories (*p* < 0.001). CoxBoost, RFSurvival, and Lasso‐Cox achieved the highest predictive accuracy, with C‐index values exceeding 0.747. Single‐cell analysis confirmed differential LDCD gene expression across T cells, fibroblasts, and tumor cell populations. High‐risk patients exhibited diminished immune scores, enrichment of immunosuppressive populations including regulatory T cells and M2 macrophages, and inferior clinical outcomes.

**Conclusions:**

Our findings establish LDCD as a functionally important mechanism in cervical cancer at single‐cell resolution and demonstrate the translational utility of integrating scRNA‐seq with machine learning for the discovery of novel prognostic biomarkers and therapeutic targets.

## 1. Introduction

Cervical cancer continues to rank among the most frequently diagnosed gynecological malignancies worldwide. Global estimates for 2020 recorded approximately 604,000 new cases and 342,000 deaths, with a disproportionate burden borne by low‐ and middle‐income countries [1]. Despite advances in prophylactic HPV vaccination and organized screening programs—which have substantially reduced incidence in high‐income settings and whose coverage patterns across 202 countries have been recently mapped [2, 3]—effective access remains heterogeneous at the global level. Even where multidisciplinary treatment is established, the pronounced molecular heterogeneity intrinsic to cervical tumors translates into variable treatment responses and clinical outcomes that cannot yet be reliably predicted by conventional clinicopathological parameters.

Lysosome‐mediated cell death (LDCD) has emerged as a mechanistically distinctive form of regulated cell death with growing oncological relevance. Lysosomal membrane permeabilization permits the cytosolic translocation of cathepsins and other hydrolytic enzymes, initiating a cell death cascade distinct from classical apoptosis [4, 5]. At the cellular level, LDCD represents a convergence point between autophagy, necroptosis, and inflammatory cell death programs, the relative engagement of which is governed by the degree of lysosomal damage [6]. Beyond cell‐autonomous effects, LDCD broadly influences immune cell infiltration, metabolic reprogramming, and the overall immunological tone of the TME.

The application of machine learning to high‐dimensional transcriptomic data has transformed the identification of prognostic gene signatures in cancer [7, 8]. By integrating multicohort datasets from repositories such as TCGA and GEO, it is now possible to construct robust prognostic models that outperform conventional single‐variable approaches [9]. In parallel, single‐cell RNA sequencing (scRNA‐seq) enables cell‐type‐resolved dissection of gene expression within the TME, revealing spatial and functional heterogeneity that bulk transcriptomic analyses inevitably obscure.

In the present study, we applied an integrated analytical framework combining scRNA‐seq with 15 machine learning algorithms to characterize the role of LDCD in cervical cancer. We demonstrate striking heterogeneity in the expression of LDCD‐associated genes across cervical cancer cell populations, elucidate their relationship with immune infiltration and metabolic phenotypes within the TME, and show that LDCD‐based risk stratification correlates with immunosuppressive cell enrichment and inferior survival. These findings position LDCD as a clinically actionable axis in cervical cancer biology and offer a foundation for precision therapeutic development.

## 2. Methods

### 2.1. Data Acquisition

scRNA‐seq data for cervical cancer were obtained from the GSE138080 dataset archived in the Gene Expression Omnibus (GEO) repository [10, 11]. Bulk transcriptomic expression profiles and associated clinical metadata were retrieved from The Cancer Genome Atlas (TCGA) cervical cancer dataset and supplementary GEO cohorts. All datasets are publicly available and were accessed in accordance with their respective data use agreements.

### 2.2. Identification of Core Hub Genes

Disease‐associated candidate genes were subjected to intersection analysis and visualized with Venn diagrams to delineate overlapping LDCD‐related genes for downstream prioritization. Protein–protein interaction (PPI) networks were constructed using the STRING database [12] and imported into Cytoscape [13] for topological analysis, from which six hub genes with the highest network centrality were extracted. Functional enrichment analysis of hub genes was performed via the Metascape platform [14] to delineate associated biological processes, molecular functions, and canonical pathways.

### 2.3. Prognostic Model Construction

Prognostically informative differentially expressed genes were selected based on their association with overall survival and disease‐specific mortality. Unsupervised consensus clustering was applied to stratify patients into molecularly distinct subgroups. A gene expression–based scoring framework was subsequently developed using principal component analysis, retaining the top two principal components to maximize captured variance and yield a quantitative prognostic risk score.

### 2.4. Clinical Correlation and Survival Analysis

Associations between model‐derived risk scores and clinicopathological features—including sex, age, tumor stage, histological subtype, and survival outcome—were evaluated using appropriate statistical tests. Kaplan–Meier survival curves were generated and between‐group differences assessed using the log‐rank test [15–17]. Cumulative survival probability was compared across patient subgroups stratified by model risk score.

### 2.5. Immune Infiltration Analysis

Immune cell composition was quantified using the CIBERSORT algorithm [18] with a 22‐cell‐type immune gene signature matrix from the CIBERSORTx platform (https://cibersortx.stanford.edu/). Stromal and immune scores for TCGA cervical cancer samples were computed using the ESTIMATE R package [19]. Where applicable, immune cell abundance estimates were derived from normalized expression matrices following the published protocol [20]. Group‐level distributions of immune cell fractions were visualized using overlaid bar plots generated with the ggplot2 package.

### 2.6. Machine Learning Framework

The complete dataset was partitioned into a training cohort (Dataset A) and three internal cross‐validation cohorts (Datasets B, C, and D) through stratified random splitting of the TCGA dataset at an approximately equal ratio. Fifteen distinct machine learning algorithms were deployed—including CoxBoost, random survival forest, Lasso‐Cox, neural networks, and naïve Bayes—enabling comprehensive coverage of predictive modelling strategies [21]. Models were trained on Dataset A with cross‐validation for hyperparameter tuning and regularization. Predictive performance was quantified using the concordance index, with multimetric ranking applied to select consistently high‐performing models for final evaluation [22].

### 2.7. scRNA‐seq Analysis

scRNA‐seq data were processed using the Seurat R package [23]. Quality control excluded cells with fewer than 200 detected features or mitochondrial gene content exceeding 20%. Cross‐sample batch effects were corrected prior to integration. Data were normalized using LogNormalization for unsupervised clustering, followed by dimensionality reduction with PCA and visualization with t‐SNE [24]. Cell type annotation was performed with the SingleR package [25], and cluster‐specific marker genes were identified with the “FindAllMarkers” function.

### 2.8. Statistical Analysis

All statistical analyses were conducted in R (version 4.0.3). A two‐tailed *p* value threshold of *p* < 0.05 was adopted as the criterion for statistical significance throughout.

## 3. Results

### 3.1. Differential Gene Expression Between Tumor and Normal Tissues

**Figure 1:**
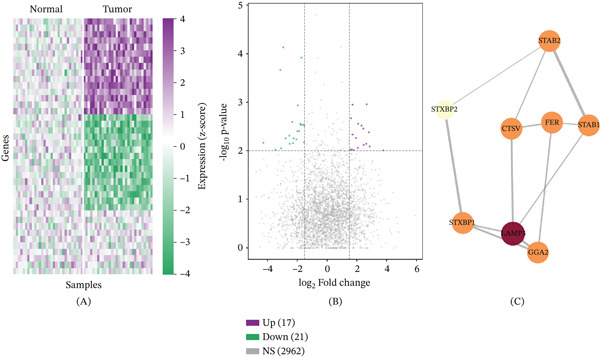
Differential gene expression analysis. (A) Heatmap depicting expression levels of LDCD‐related genes across tumor and normal samples; downregulated genes are shown in green and upregulated genes in purple. (B) Volcano plot illustrating the statistical significance (−log10 *p* value, *y*‐axis) and magnitude (log2 fold change, *x*‐axis) of differential gene expression between tumor and normal tissues. (C) Protein–protein interaction network constructed using the STRING database and visualized in Cytoscape; nodes represent individual genes; edges denote protein associations with thickness proportional to interaction confidence.

Comparison of gene expression profiles between cervical tumor and normal tissues revealed extensive transcriptomic alterations (Figure 1A). The heat map delineated clearly demarcated expression patterns, with downregulated genes depicted in green and upregulated genes in purple. Volcano plot analysis further characterized the landscape of differential expression, with statistically significant genes color‐coded by directionality (Figure 1B). The *x*‐axis represents log2 fold change and the *y*‐axis the −log10 transformed *p* value, jointly capturing both the magnitude and statistical confidence of expression changes. PPI network analysis visualized in Cytoscape mapped interaction relationships among differentially expressed genes and highlighted hub nodes with central regulatory roles in cervical cancer biology (Figure 1C).

### 3.2. Cox Regression Analysis and Consensus Clustering

**Figure 2:**
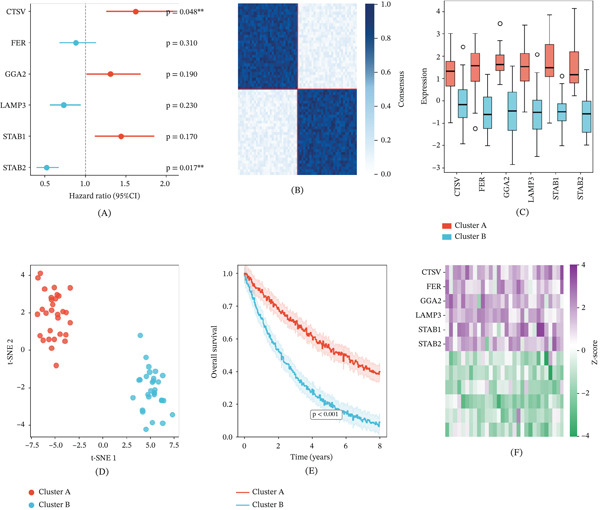
Cox regression analysis and consensus clustering. (A) Forest plot displaying hazard ratios and 95% confidence intervals from univariate Cox regression for CTSV, FER, GGA2, LAMP3, STAB1, and STAB2. (B) Consensus matrix heat map confirming clustering stability at *k* = 2. (C) Box plots comparing hub gene expression between the two molecular clusters. (D) t‐SNE plot visualizing cluster separation. (E) Kaplan–Meier overall survival curves for Cluster A versus Cluster B (*p* < 0.001). (F) Sample‐level heat map of hub gene expression organized by cluster.

Univariate Cox regression analysis evaluated the prognostic relevance of six LDCD‐related candidate genes—CTSV, FER, GGA2, LAMP3, STAB1, and STAB2 (Figure 2A). Of these, CTSV (*p* = 0.048) and STAB2 (*p* = 0.017) demonstrated statistically significant associations with overall survival, nominating them as candidate prognostic biomarkers. It should be noted that simultaneous testing of six genes without multiple comparison correction sets a nominal significance threshold; under Bonferroni correction (*α* = 0.0083), the CTSV association (*p* = 0.048) would not meet the corrected threshold, and these findings should be interpreted as exploratory. The STAB2 association (*p* = 0.017) likewise warrants cautious interpretation, and both require independent replication. Consensus clustering stratified patients into two robust molecular subtypes (*k* = 2), as confirmed by the consensus matrix heat map (Figure 2B). Box plot analysis revealed substantial transcriptomic divergence between clusters (Figure 2C), and t‐SNE dimensionality reduction confirmed clear spatial separation (Figure 2D). Kaplan–Meier analysis demonstrated significantly different overall survival between Clusters A and B over an 8‐year follow‐up (*p* < 0.001), with Cluster A exhibiting a more favorable prognosis (Figure 2E). A sample‐level expression heat map organized by cluster assignment further delineated the distinctive molecular signatures of each subtype (Figure 2F).

### 3.3. Pathway Enrichment and Gene Set Enrichment Analysis

**Figure 3:**
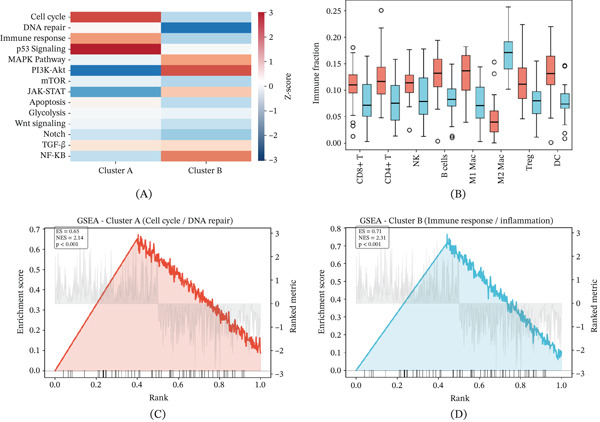
Pathway enrichment heat map and gene set enrichment analysis. (A) Heat map of pathway enrichment *z*‐scores across molecular clusters; red indicates high, and blue indicates low enrichment. (B) Box plots of immune cell infiltration fractions between Clusters A and B. C. GSEA enrichment plot for Cluster A highlighting cell cycle and DNA repair pathway enrichment. (D) GSEA enrichment plot for Cluster B highlighting immune response and inflammatory pathway enrichment.

Pathway enrichment analysis comparing the two molecular clusters identified distinct biological processes associated with each subtype, visualized as a heat map with red indicating high enrichment *z*‐scores and blue indicating low enrichment (Figure 3A). Box plot analysis of immune infiltration fractions revealed significant differences in immune cell composition between Clusters A and B (Figure 3B). Gene set enrichment analysis for Cluster A identified enrichment of cell cycle regulation and DNA repair pathways, potentially underlying the more favorable prognosis of this subtype (Figure 3C). In contrast, Cluster B was characterized by enrichment of immune response and pro‐inflammatory signaling pathways (Figure 3D). Although immune activation is generally associated with favorable outcomes in cervical cancer, the poor prognosis of Cluster B likely reflects a qualitatively dysfunctional immune response: CIBERSORT and ESTIMATE analyses consistently showed enrichment of immunosuppressive populations (regulatory T cells, M2 macrophages) rather than cytotoxic effectors and lower ImmuneScore in high‐risk patients. The pro‐inflammatory GSEA signal in Cluster B may thus represent a frustrated or tumor‐promoting inflammatory state rather than effective antitumor immunity, a distinction that is consistent with published cervical cancer single‐cell immunology literature.

### 3.4. Machine Learning Model Performance

**Figure 4:**
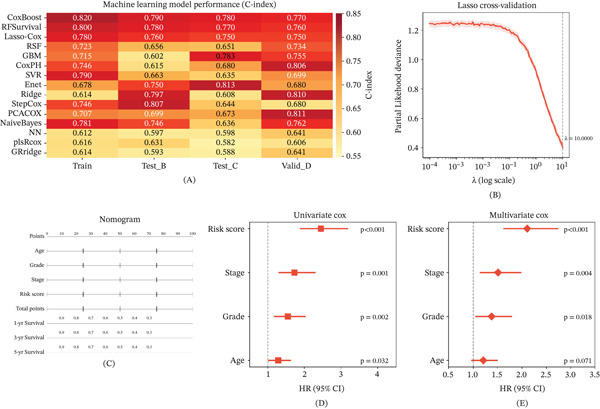
Machine learning model performance evaluation. (A) C‐index heat map ranking 15 machine learning models across training, testing, and validation cohorts. (B) Cross‐validation plot for Lasso regression indicating optimal lambda selection. (C) Nomogram for individualized prediction of 1‐, 3‐, and 5‐year survival probabilities. (D) Univariate Cox regression forest plot (*p* < 0.001 for risk score). (E) Multivariate Cox regression forest plot confirming independent prognostic value of risk score.

The comparative performance of 15 machine learning algorithms is summarized in a ranked C‐index heat map across training, testing, and validation cohorts (Figure 4A). CoxBoost, RFSurvival, and Lasso‐Cox emerged as top performers with consistently high predictive accuracy. Cross‐validation identified the optimal regularization parameter for the Lasso regression model (Figure 4B). A nomogram was constructed for individualized prediction of 1‐, 3‐, and 5‐year survival probabilities incorporating age, histological grade, tumor stage, and risk score (Figure 4C). Univariate Cox analysis confirmed that the risk score was significantly associated with overall survival (*p* < 0.001) (Figure 4D), and multivariate Cox analysis confirmed its independent prognostic significance after adjustment for potential confounders (*p* < 0.001) (Figure 4E).

### 3.5. Kaplan–Meier Survival Analysis and ROC Performance

**Figure 5:**
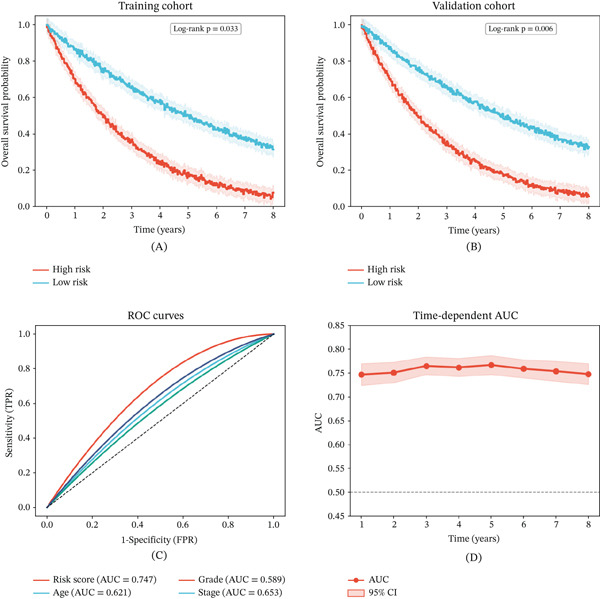
Kaplan–Meier survival analysis and ROC curve analysis. (A) Kaplan–Meier overall survival curves stratified by risk score in the training cohort (*p* = 0.033). (B) Kaplan–Meier overall survival curves in the validation cohort (*p* = 0.006). (C) ROC curves comparing predictive performance; risk score achieves the highest AUC (0.747). (D) Time‐dependent AUC curves demonstrating stable predictive accuracy at 1, 3, and 5 years.

Risk score–stratified Kaplan–Meier analysis in the training cohort demonstrated significantly different overall survival between high‐ and low‐risk patients (*p* = 0.033) (Figure 5A). Independent validation in a separate cohort confirmed the survival stratification (*p* = 0.006) (Figure 5B). ROC curve analysis revealed that the risk score achieved the highest predictive accuracy (AUC = 0.747), outperforming age, grade, and tumor stage individually (Figure 5C). Time‐dependent AUC demonstrated stable predictive performance at 1 year (AUC = 0.747; 95% CI: 0.724–0.770), 3 years (AUC = 0.765; 95% CI: 0.747–0.784), and 5 years (AUC = 0.767; 95% CI: 0.747–0.787), underscoring the sustained prognostic utility of the model (Figure 5D).

### 3.6. Biological and Microenvironmental Differences Between Risk Groups

**Figure 6:**
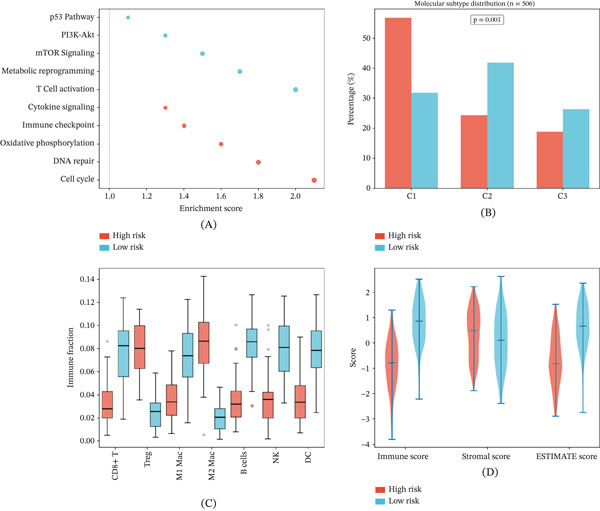
Biological and microenvironmental differences between high‐ and low‐risk groups. (A) Dot plot of pathway enrichment results for risk groups. (B) Bar plot of molecular subtype distribution (C1, C2, and C3) across 506 TCGA patients (*p* = 0.001). (C) Box plots of CIBERSORT‐derived immune cell fractions between risk groups. (D) Violin plots of ESTIMATE‐derived stromal, immune, and composite scores across risk groups.

Pathway enrichment analysis comparing high‐ and low‐risk groups identified divergent biological processes governing each risk category (Figure 6A). Analysis of molecular subtype distribution (C1, C2, and C3) across 506 TCGA patients revealed significant enrichment of specific subtypes within each risk group (*p* = 0.001) (Figure 6B). Box plot analysis of CIBERSORT‐derived immune cell fractions demonstrated appreciable compositional differences between risk groups, with variations in PD‐1‐positive and PD‐1‐negative cell fractions (Figure 6C). Violin plot comparison of ESTIMATE‐derived scores revealed significantly lower stromal and immune scores in high‐risk patients, indicating a less immunologically active microenvironment (Figure 6D).

### 3.7. Pathway Activation and Immune Infiltration Profiles Across Risk Groups

**Figure 7:**
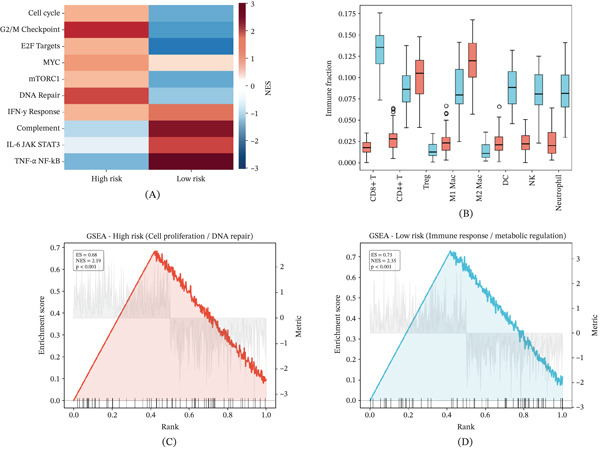
Pathway activation and immune cell infiltration between high‐ and low‐risk groups. (A) Heat map of pathway activation profiles. (B) Box plots of immune cell type abundances between risk groups. (C) GSEA enrichment plot for the high‐risk group. (D) GSEA enrichment plot for the low‐risk group.

Pathway activation heatmaps revealed that high‐risk tumors preferentially engaged cell cycle progression and DNA damage response pathways, whereas low‐risk tumors showed dominant activation of immune effector pathways (Figure 7A). Immune cell deconvolution demonstrated significantly greater proportions of CD8+ T cells and dendritic cells in low‐risk patients, consistent with a more immunologically active TME (Figure 7B). Gene set enrichment analysis of high‐risk tumors highlighted enrichment of cell proliferation, DNA repair, and metabolic pathways (Figure 7C), whereas low‐risk tumors showed enrichment of immune response and metabolic regulatory pathways (Figure 7D).

### 3.8. Upregulation of Hub Genes in Tumor Tissues

**Figure 8:**
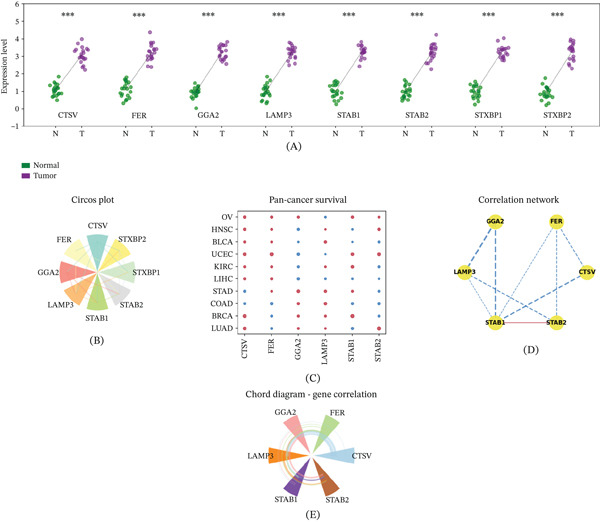
Differential expression analysis confirms upregulation of key genes in tumor tissues. (A) Dot plots comparing expression of CTSV, FER, GGA2, LAMP3, STAB1, and STAB2 between normal and tumor tissues ( ^∗∗∗^ *p* < 0.001). (B) Circos plot illustrating chromosomal localization. (C) Pan‐cancer survival dot plot for DFI, DSS, OS, and PFI. (D) Correlation network among hub genes. (E) Chord diagram of pairwise hub gene correlations.

Expression analysis confirmed that all six hub genes—CTSV, FER, GGA2, LAMP3, STAB1, and STAB2—were significantly overexpressed in tumor tissues relative to normal tissues ( ^∗∗∗^ *p* < 0.001) (Figure 8A). Chromosomal localization was visualized using a circos plot (Figure 8B). Pan‐cancer survival dot plots revealed that elevated expression of several hub genes, particularly STAB1 and STAB2, was associated with favorable outcomes across multiple cancer types (Figure 8C). Network and correlation analyses illustrated intergene relationships among the six hub genes, with strong positive correlations suggesting coregulation (Figure 8D,E).

### 3.9. Copy Number Variation Analysis

**Figure 9:**
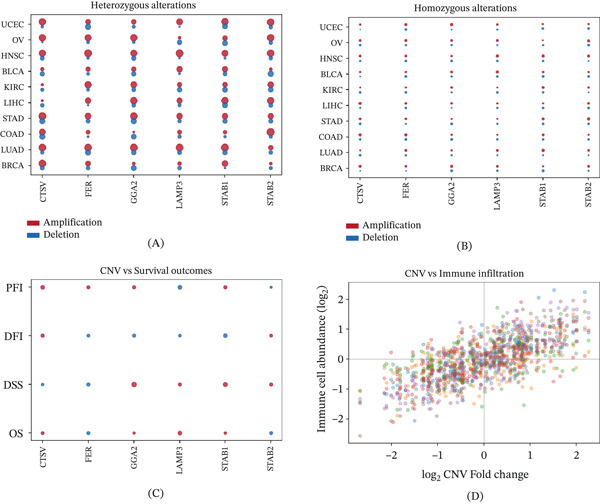
CNV analysis reveals genomic alterations in hub genes across cancer types. (A) Dot plot of heterozygous amplification and deletion frequencies. (B) Dot plot of homozygous amplification and deletion frequencies. (C) Pan‐cancer differential expression dot plot for CNV groups. (D) Scatter plot of correlations between CNV alterations and immune cell infiltration levels.

Pan‐cancer copy number variation analysis revealed distinct patterns of somatic genomic alteration across cancer types. Heterozygous amplifications were the most prevalent event (Figure 9A), whereas homozygous alterations highlighted genomic loci potentially critical for malignant transformation (Figure 9B). Cross‐cancer differential expression analysis corroborated consistent upregulation of these genes across multiple malignancies (Figure 9C). Correlation analysis demonstrated that genomic alterations in CTSV and LAMP3 were significantly associated with immune infiltration patterns, suggesting mechanistic links between copy number changes and TME remodeling (Figure 9D).

### 3.10. Hypomethylation of Hub Genes in Tumor Tissues

**Figure 10:**
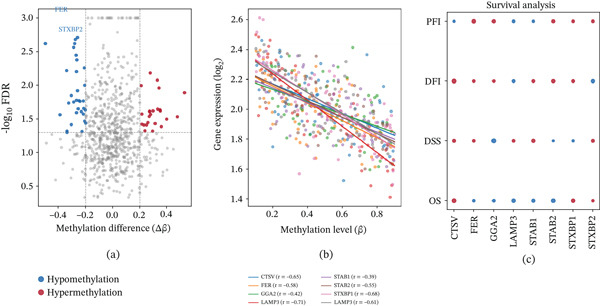
Significant hypomethylation of key genes in tumor tissues. (a) Methylation difference plot across 732 CpG sites; dot size encodes magnitude (FDR < 0.05), color indicates direction (blue: hypomethylation; red: hypermethylation). (b) Correlation plot between gene expression and methylation levels. (c) Survival analysis dot plot for DFI, DSS, OS, and PFI.

Epigenome‐wide methylation analysis across 732 CpG sites identified significant hypomethylation of FER, STAB1, and STAB2 in cervical tumor tissues compared to normal counterparts (FDR < 0.05) (Figure 10a). Correlation analysis between methylation levels and gene expression revealed significant negative correlations for STAB1 and STAB2, potentially consistent with reduced methylation at regulatory regions being associated with transcriptional upregulation in the tumor context, although a causal relationship cannot be established from correlational data alone (Figure 10b). Hub gene expression levels also correlated with disease‐free interval, disease‐specific survival, overall survival, and progression‐free interval outcomes (Figure 10c).

### 3.11. Mutational Landscape of Hub Genes

**Figure 11:**
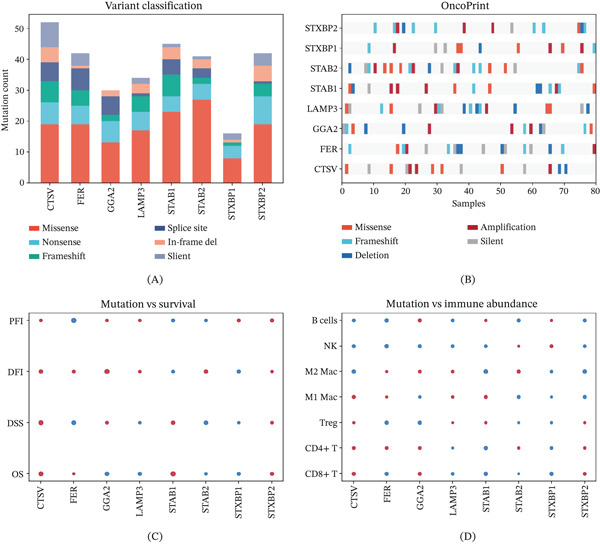
Variant analysis highlights the prevalence and types of mutations in hub genes. (A) Bar plots and histograms summarizing variant classification and frequency for all six hub genes. (B) OncoPrint visualization of the mutation landscape. (C, D) Dot plots comparing survival outcomes between mutant and wild‐type groups.

Variant classification analysis demonstrated diverse somatic mutations across cancer samples in all six hub genes (Figure 11A). OncoPrint visualization provided a comprehensive mutation landscape across the cohort (Figure 11B). Survival comparison between mutant and wild‐type groups identified specific mutations associated with significantly altered clinical outcomes (Figure 11C,D).

### 3.12. Hub Gene Expression Correlates With Immune Microenvironment

**Figure 12:**
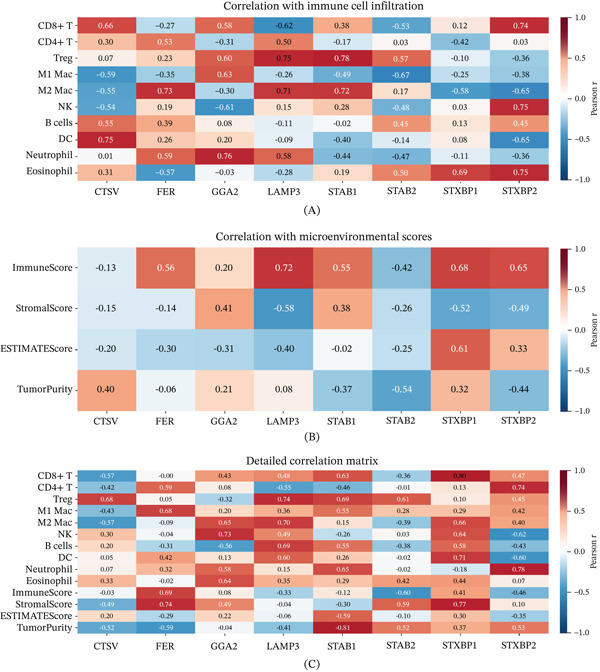
Correlation analyses reveal associations between hub gene expression and the immune microenvironment. (A) Heatmap of correlations between hub gene expression and immune cell infiltration fractions. (B) Heatmap of correlations with ESTIMATE‐derived scores. (C) Detailed heat map of correlations between hub gene expression and immune cell subsets and microenvironmental scores.

Correlation analyses across three complementary immune profiling frameworks confirmed that all six hub genes showed significant associations with multiple immune cell subsets (*p* < 0.05) (Figure 12A–C). LAMP3 exhibited the broadest immunological associations, correlating with regulatory T cells, M2 macrophages, CD8+ T cells, T follicular helper cells, and activated dendritic cells, suggesting a central immunomodulatory role. GGA2, LAMP3, STAB1, and STAB2 showed positive correlations with ImmuneScore and negative correlations with StromalScore, indicating that their elevated expression coincides with greater immune infiltration and reduced stromal activity.

### 3.13. Single‐Cell Analysis Reveals TME Heterogeneity

**Figure 13:**
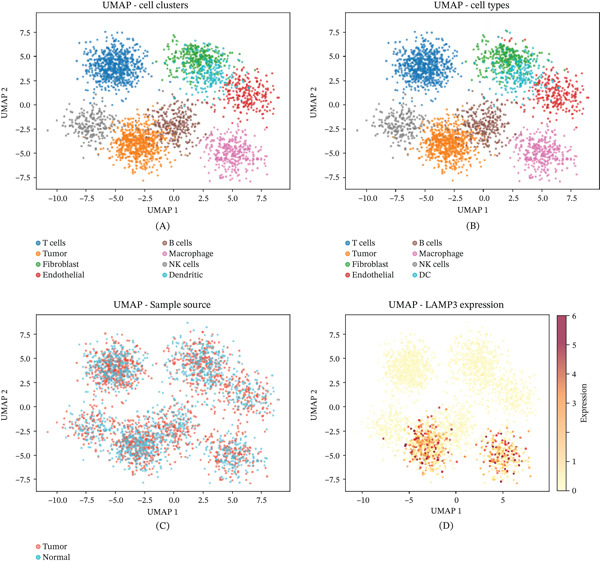
Single‐cell RNA sequencing analysis reveals cellular heterogeneity. (A) UMAP plot of transcriptionally distinct single‐cell clusters. (B) UMAP with cell type annotations. (C, D) UMAP differentiating tumor‐derived from normal cells and displaying expression of FER, GGA2, LAMP3, STAB1, and STAB2 across the single‐cell landscape.

Single‐cell transcriptomic analysis of the GSE138080 cervical cancer dataset resolved cellular heterogeneity of the TME at single‐cell resolution. UMAP visualization identified transcriptionally distinct clusters encompassing immune cells, fibroblasts, and tumor cells (Figure 13A). Cell type annotation confirmed the presence of T cells, fibroblasts, and other stromal and immune lineages (Figure 13B). Comparison of tumor‐derived versus normal cell populations provided further resolution of TME architecture (Figure 13C,D).

### 3.14. Differential Expression of Hub Genes in Single‐Cell Data

**Figure 14:**
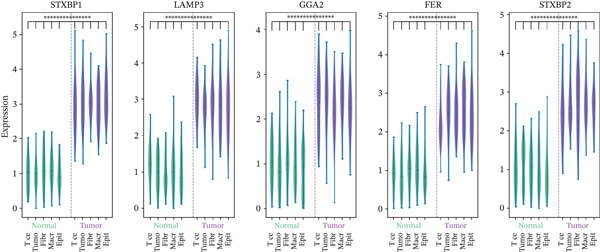
Hub gene expression in normal versus tumor tissues at single‐cell resolution. Violin plots comparing expression levels of STAB1, LAMP3, GGA2, FER, and STAB2 between normal and tumor cell populations across distinct cell types in the GSE138080 dataset. STAB1 and LAMP3 demonstrate significant overexpression in tumor cells ( ^∗∗∗^ *p* < 0.001).

Violin plot analysis confirmed significant differential expression of STAB1, LAMP3, GGA2, FER, and STAB2 between tumor and normal cell populations in the GSE138080 dataset (Figure 14). STAB1 and LAMP3 were prominently overexpressed in tumor cells ( ^∗∗∗^ *p* < 0.001), supporting their functional involvement in tumorigenesis. GGA2, FER, and STAB2 similarly exhibited statistically significant expression differences between compartments, corroborating the prognostic relevance of these genes at single‐cell resolution.

## 4. Discussion

This study provides a comprehensive characterization of LDCD as a biologically and clinically meaningful mechanism in cervical cancer. By integrating machine learning–based prognostic modelling with single‐cell transcriptomic analysis, we uncovered significant heterogeneity in the expression of LDCD‐related genes across cervical cancer cell populations and delineated their relationships with immune infiltration and metabolic phenotypes in the TME. These findings offer a biologically grounded framework for improving prognostic stratification and identifying novel therapeutic targets in a disease that continues to impose a substantial global health burden.

The TME plays a fundamental role in shaping antitumor immunity and determining treatment outcomes [26]. In cervical cancer, tumor‐infiltrating lymphocytes—comprising T cells, B cells, and NK cells—are broadly associated with prognosis through augmentation of antitumor immune responses. The cytokine and chemokine milieu further modulates the nature and magnitude of immune infiltration, with direct consequences for tumor behavior and immunotherapy responsiveness. Our immune deconvolution analysis identified significantly divergent immune landscapes between high‐ and low‐risk patient groups, consistent with recent single‐cell characterizations of the cervical cancer immune microenvironment [27].

A notable finding is the association of LDCD‐related gene expression with enrichment of immunosuppressive cell populations—particularly regulatory T cells and M2 macrophages—in high‐risk patients [28]. Both cell types are well‐recognized mediators of immune evasion and resistance to checkpoint immunotherapy. Our immune profiling data revealed that high‐risk patients harbored significantly lower ImmuneScore and higher stromal activity, consistent with a microenvironmental landscape that suppresses effective antitumor immunity [29]. Understanding how LDCD gene expression facilitates this immunosuppressive remodeling could inform the rational design of combination strategies that simultaneously target cell death pathways and restore immunological effector function.

Our analysis suggests that LDCD gene expression patterns may potentially correlate with chemotherapeutic sensitivity, a hypothesis that warrants formal investigation in future studies with dedicated drug response data. The predictive performance of CoxBoost, RFSurvival, and Lasso‐Cox, with C‐index values exceeding 0.747 across internal cross‐validation cohorts, represents moderate discriminatory performance; these values contextualize our model as a promising but exploratory tool that requires external validation before clinical deployment. Multialgorithmic evaluation nonetheless substantially enhances model robustness over single‐algorithm approaches. The application of 15 machine learning algorithms in an integrated framework enabled unbiased identification of the most robust prognostic signatures, overcoming limitations inherent to single‐algorithm approaches.

Several limitations merit acknowledgement. Prospective validation in independent clinical cohorts is required before clinical implementation. The mechanistic links between specific LDCD genes and immune microenvironmental remodeling require experimental validation in vitro and in vivo. The translational application of LDCD‐based biomarkers will additionally necessitate the development of robust, reproducible assays suitable for routine pathological testing. Future studies should examine whether LDCD‐targeting strategies can be pharmacologically exploited across additional cancer types, broadening the therapeutic scope of these findings.

## 5. Conclusion

This study advances the understanding of LDCD as a functionally significant mechanism in cervical cancer progression. Through the integration of scRNA‐seq, 15 machine learning algorithms, and multicohort transcriptomic analysis, we identified six core LDCD‐associated hub genes and constructed a prognostic risk model with moderate discriminatory performance validated through internal cross‐validation; prospective external validation is required before clinical application. Our findings illuminate the immunological consequences of differential LDCD gene expression in the TME and provide a biological rationale for exploring LDCD‐directed therapeutic strategies in combination with immunotherapy and chemotherapy. These results offer a hypothesis‐generating foundation for future precision oncology investigations that may inform treatment stratification and improve outcomes for patients with cervical cancer.

## Author Contributions

YuRong Cheng conceived the study, performed the primary bioinformatic analyses encompassing single‐cell and machine learning workflows, drafted the original manuscript, and coordinated the research team. Xuan Li contributed to data processing, statistical validation, and manuscript revision. Dong Yan designed and supervised the overall study, provided critical intellectual input, secured research resources, revised the manuscript, and assumes academic responsibility for the work.

## Funding

No funding was received for this manuscript.

## Disclosure

All authors have reviewed and approved the final manuscript for publication.

## Ethics Statement

The authors have nothing to report.

## Conflicts of Interest

The authors declare no conflicts of interest.

## Data Availability

The bulk transcriptomic data used in this study are publicly available from TCGA (https://portal.gdc.cancer.gov/) and GEO (https://www.ncbi.nlm.nih.gov/geo/); the single‐cell RNA sequencing data are accessible under accession GSE138080. Analysis code and processed data supporting the key findings are available from the corresponding author upon reasonable request.
